# How to Spell and Pronounce Aluminium

**Published:** 1871-12

**Authors:** W. E. Driscoll


### HOW TO SPELL AND PRONOUNCE ALUMINIUM.

BY W. E. DRISCOLL.

An incorrect spelling or pronunciation of words in common use is not calculated to establish a reputation for good scholarship. All have noticed, no doubt, that for a few years past almost every writer in the dental papers has spelled aluminium without the second "i." Now, if this was only a matter of mere taste or sound, or of easy pronunciation, it might be very well. But there is another consideration, of more importance than either of the above. It probably is not generally known that there is a general agreement among scientific societies in Europe to spell all names of metals that are found in the metallic state with "um," (if the word can be so terminated) omitting the "i" before it; and those metals that are not found in the metallic form, but depend upon chemical art to bring them to this state, are spelled with the "i" before the "um" as the last syllable, thus affording easy means of distinguishing, both by the spelling and the pronunciation, whether a metal is "chemical" or not.

This, certainly, constitutes sufficient reason for continuing the two forms of spelling; and as they may be continued, should there not be some intelligible rule for their use? And if so, what better one than the one here insisted upon?

